# Supplementary material for: Rarefaction is better than robust Aitchison PCA and other compositional data analysis methods at controlling for uneven sequencing effort
Source: mSystems. 2026 Sep 30;11(10):e00930-26. doi: 10.1128/msystems.00930-26 (PMC13712867; doi:10.1128/msystems.00930-26)
Supplement: Supplementary file 1 [file msystems.00930-26-s0001.pdf]

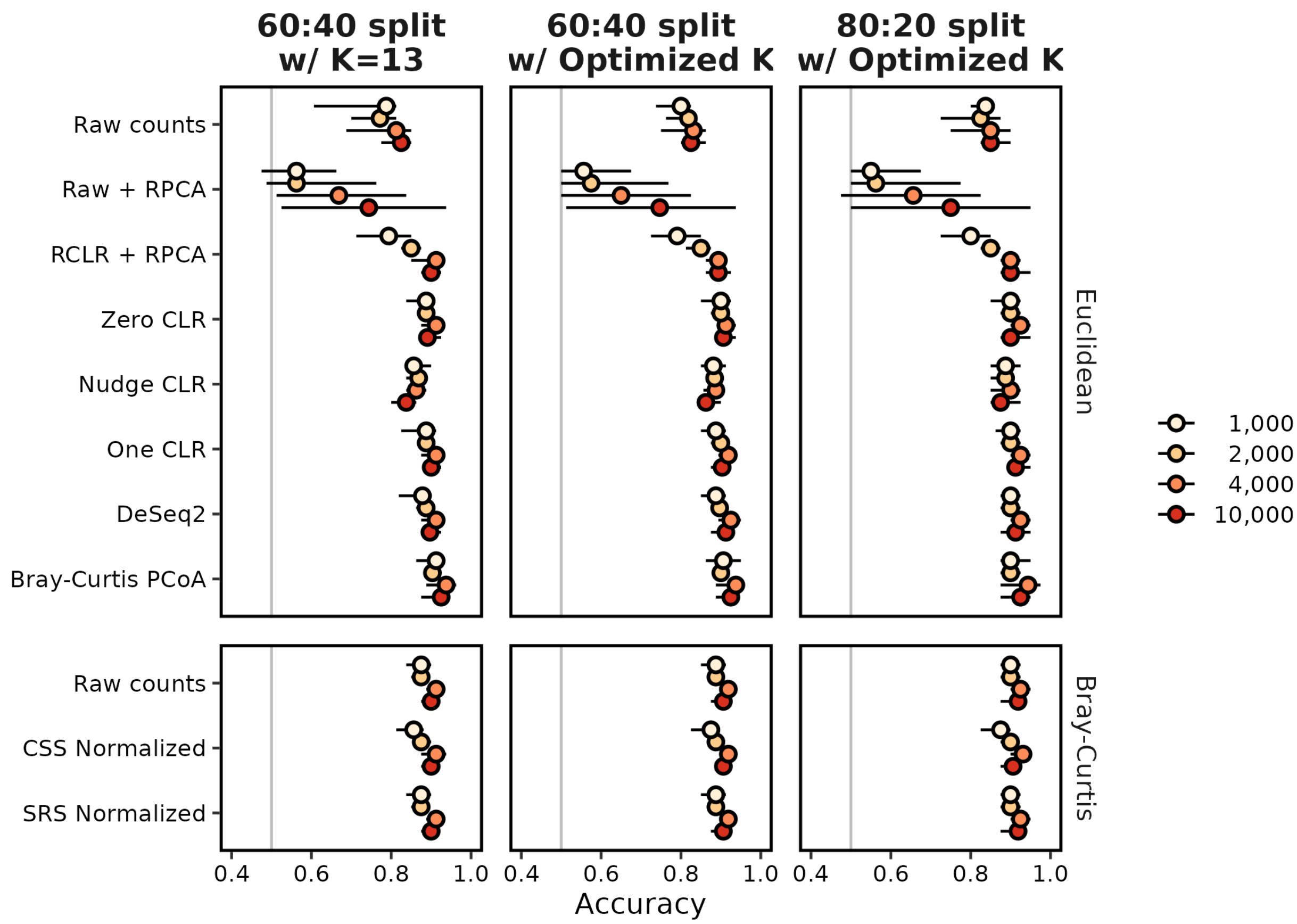

Figure S1. The choice of K and the size of the training and testing data sets had a minimal effect on KNN classification accuracy when using the original Martino et al. overlap simulation data. Martino et al. split 60% of their data into a training set using 13 neighbors and tested on the remaining 40%. Following Topcuoglu et al. (28), I optimized the number of neighbors using 60:40 and 80:20 training-testing splits. The 80:20 data were used in the Results section.
