# Supplementary material for: Rarefaction is better than robust Aitchison PCA and other compositional data analysis methods at controlling for uneven sequencing effort
Source: mSystems. 2026 Sep 30;11(10):e00930-26. doi: 10.1128/msystems.00930-26 (PMC13712867; doi:10.1128/msystems.00930-26)
Supplement: Supplementary file 4 [file reviewer-comments.pdf]

ASM | mSystems

Rarefaction is better than robust Aitchison PCA and other compositional data analysis methods at controlling for uneven sequencing effort

Patrick Schloss

Corresponding Author(s): Patrick Schloss, University of Michigan

Review Timeline:

	Submission Date:	July 17, 2026
	Editorial Decision:	August 6, 2026
	Revision Received:	August 7, 2026
	Accepted:	August 31, 2026

Editor: Mark Mandel

Reviewer(s): The reviewers have opted to remain anonymous.

Transaction Report:

(Note: With the exception of the correction of typographical or spelling errors that could be a source of ambiguity, letters and reports are not edited. The original formatting of letters and referee reports may not be reflected in this compilation.)

DOI: https://doi.org/10.1128/msystems.00930-26

Re: mSystems00930-26 (Rarefaction is better than robust Aitchison PCA and other compositional data analysis methods at controlling for uneven sequencing effort)

Dear Dr. Patrick D. Schloss:

Thank you for the privilege of reviewing your work. I am sending this correspondence in place of Editor Susannah Tringe who is on vacation, but who did not want your manuscript to be delayed. We discussed your submission and feel that the work provides a valuable contribution to the field, addresses the methodological concerns raised by the reviewers, and highlights the importance of open data and rigorous independent evaluation of scientific advances. Given that your manuscript is a direct rebuttal to a previous paper, we think that a "Matters Arising" piece is the best format for the work. To facilitate that process, we will not restrict the work to the typical word and figure limits for these pieces given the thoroughness by which you have presented your arguments.

The one minor edit we will request is to reference the previous paper as "Martino et al." rather than "Martino", to focus on the group of authors rather than to single out an individual author. I would ask you to please resubmit the manuscript with this edit throughout your piece. If these changes are the only revisions, then only the manuscript document needs to be uploaded for the revision. If any other changes are made, please also include the marked-up manuscript. ASM staff will convert your submission to the "Matters Arising" format; no action on your part is required for the article type update.

With your manuscript ready for acceptance, and consistent with ASM Journals handling of these pieces, we will invite a response from the authors of the original study to be published alongside your study.

In your response document you noted that you would consider a "Matters Arising" format if the length could accommodate your analysis. We therefore hope that this proposed path is satisfactory, and we very much look forward to your resubmitted manuscript. If you have any questions, please reach out to Dr. Tringe and myself.

Additionally, I want to be sure to convey that your concerns about AI reviews were taken seriously and were considered by the handling editor at the time. There was no evidence that generative AI was used in formation of the ideas in the reviews. As an aside, I felt that both of the reviewers raised substantive issues and I think that they provided valuable inputs to consider.

Please return the manuscript within 60 days; if you cannot complete the modification within this time period, please contact me. If you do not wish to modify the manuscript and prefer to submit it to another journal, notify me immediately so that the manuscript may be formally withdrawn from consideration by mSystems.

Revision Guidelines
To submit your modified manuscript, log into the submission site at https://msystems.msubmit.net/cgi-bin/main.plex. Go to Author Tasks and click the appropriate manuscript title to begin. The information you entered when you first submitted the paper will be displayed; update this as necessary. Note the following requirements:

• Upload point-by-point responses to the issues raised by the reviewers in a file named "Response to Reviewers," NOT in your cover letter.
• Upload a compare copy of the manuscript (without figures) as a "Marked-Up Manuscript" file.
• Upload a clean .DOC/.DOCX version of the revised manuscript and remove the previous version.
• Each figure must be uploaded as a separate, editable, high-resolution file (TIFF or EPS preferred), and any multipanel figures must be assembled into one file.
• Any supplemental material intended for posting by ASM should be uploaded with their legends separate from the main manuscript. You can combine all supplemental material into one file (preferred) or split it into a maximum of 10 files with all associated legends included.

For complete guidelines on revision requirements, see our Submission and Review Process webpage. Submission of a paper that does not conform to guidelines may delay acceptance of your manuscript.

Data availability: ASM policy requires that data be available to the public upon online posting of the article, so please verify all links to sequence records, if present, and make sure that each number retrieves the full record of the data. If a new accession number is not linked or a link is broken, provide mSystems production staff with the correct URL for the record. If the accession numbers for new data are not publicly accessible before the expected online posting of the article, publication may be delayed; please contact production staff (mSystems@asmusa.org) immediately with the expected release date.

Publication Fees: For information on publication fees and which article types are subject to charges, visit ourwebsite. If your manuscript is accepted for publication and any fees apply, you will be contacted separately about payment during the production process; please follow the instructions in that e-mail. Arrangements for payment must be made before your article is published.

ASM Membership: Corresponding authors may join or renew ASM membership to obtain discounts on publication fees. Need to upgrade your membership level? Please contact Customer Service at Service@asmusa.org.

The ASM Journals program strives for constant improvement in our submission and publication process. Please tell us how we can improve your experience by taking this quick Author Survey.

Thank you for submitting your paper to mSystems.

Sincerely,
Mark Mandel
Senior Editor
mSystems

Re: mSystems00930-26R1 (Rarefaction is better than robust Aitchison PCA and other compositional data analysis methods at controlling for uneven sequencing effort)

Dear Dr. Patrick D. Schloss:

Your manuscript has been accepted, and I am forwarding it to the ASM production staff for publication. Your paper will first be checked to make sure all elements meet the technical requirements. ASM staff will contact you if anything needs to be revised before copyediting and production can begin. Otherwise, you will be notified when your proofs are ready to be viewed.

Data Availability: ASM policy requires that data be available to the public upon online posting of the article, so please verify all links to sequence records, if present, and make sure that each number retrieves the full record of the data. If a new accession number is not linked or a link is broken, provide production staff with the correct URL for the record. If the accession numbers for new data are not publicly accessible before the expected online posting of the article, publication may be delayed; please contact ASM production staff immediately with the expected release date.

Publication Fees: For information on publication fees and which article types have charges, please visit our website. We have partnered with Copyright Clearance Center (CCC) to collect author charges. If fees apply to your paper, you will receive a message from no-reply@copyright.com with further instructions. For questions related to paying charges through RightsLink, please contact CCC at ASM_Support@copyright.com or toll free at +1-877-622-5543. CCC makes every attempt to respond to all emails within 24 hours.

ASM Membership: Corresponding authors may join or renew ASM membership to obtain discounts on publication fees. Need to upgrade your membership level? Please contact Customer Service at Service@asmusa.org.

Embargo Policy: A press release may be issued as soon as the manuscript is posted on the mSystems Latest Articles webpage. The corresponding author will receive an email with the subject line "ASM Journals Author Services Notification" when the article is available online.

Cover Image Submissions: If you would like to submit a potential Cover Image, please email a file and a short legend to msystems@asmusa.org. Please note that we can only consider images that (i) the authors created or own and (ii) have not been previously published. By submitting, you agree that the image can be used under the same terms as the published article. Image File requirements: TIF/EPS, 7.5 inches wide by 8.25 inches tall (at least 2,250 pixels wide by 2,475 pixels tall), minimum 300 dpi resolution (600 dpi preferred), RGB, and no figure elements, e.g., arrows or panel labels. The legend should be a short description of the image, 1-2 sentences recommended. Please use the ASM Journals Cover Image Sizing Tool (https://journals.asm.org/pb-assets/html/asm-cover-sizing-tool.html) to ensure that your proposed cover image meets our size requirements.

Thank you for submitting your paper to mSystems.

Sincerely,
Mark Mandel
Editor
mSystems
